# Stoichiometry of Base Excision Repair Proteins Correlates with Increased Somatic CAG Instability in Striatum over Cerebellum in Huntington's Disease Transgenic Mice

**DOI:** 10.1371/journal.pgen.1000749

**Published:** 2009-12-04

**Authors:** Agathi-Vassiliki Goula, Brian R. Berquist, David M. Wilson, Vanessa C. Wheeler, Yvon Trottier, Karine Merienne

**Affiliations:** 1 Department of Neurobiology and Genetics, Institute of Genetics and Molecular and Cellular Biology (IGBMC), UMR 7104-CNRS/INSERM/UdS, Illkirch, France; 2 Laboratory of Molecular Gerontology, National Institute on Aging (NIA)/National Institutes of Health (NIH), Baltimore, Maryland, United States of America; 3 Center for Human Genetic Research, Massachusetts General Hospital, Boston, Massachussetts, United States of America; The Hospital for Sick Children and University of Toronto, Canada

## Abstract

Huntington's disease (HD) is a progressive neurodegenerative disorder caused by expansion of an unstable CAG repeat in the coding sequence of the Huntingtin (*HTT*) gene. Instability affects both germline and somatic cells. Somatic instability increases with age and is tissue-specific. In particular, the CAG repeat sequence in the striatum, the brain region that preferentially degenerates in HD, is highly unstable, whereas it is rather stable in the disease-spared cerebellum. The mechanisms underlying the age-dependence and tissue-specificity of somatic CAG instability remain obscure. Recent studies have suggested that DNA oxidation and OGG1, a glycosylase involved in the repair of 8-oxoguanine lesions, contribute to this process. We show that in HD mice oxidative DNA damage abnormally accumulates at CAG repeats in a length-dependent, but age- and tissue-independent manner, indicating that oxidative DNA damage alone is not sufficient to trigger somatic instability. Protein levels and activities of major base excision repair (BER) enzymes were compared between striatum and cerebellum of HD mice. Strikingly, 5′-flap endonuclease activity was much lower in the striatum than in the cerebellum of HD mice. Accordingly, Flap Endonuclease-1 (FEN1), the main enzyme responsible for 5′-flap endonuclease activity, and the BER cofactor HMGB1, both of which participate in long-patch BER (LP–BER), were also significantly lower in the striatum compared to the cerebellum. Finally, chromatin immunoprecipitation experiments revealed that POLβ was specifically enriched at CAG expansions in the striatum, but not in the cerebellum of HD mice. These *in vivo* data fit a model in which POLβ strand displacement activity during LP–BER promotes the formation of stable 5′-flap structures at CAG repeats representing pre-expanded intermediate structures, which are not efficiently removed when FEN1 activity is constitutively low. We propose that the stoichiometry of BER enzymes is one critical factor underlying the tissue selectivity of somatic CAG expansion.

## Author Summary

Huntington's disease (HD) is a neurodegenerative disorder that belongs to a family of genetic diseases caused by abnormal expansion of CAG/CTG repetitive sequences. The instability of trinucleotide repeat expansions in germline and somatic cells has deleterious clinical consequences in HD. For instance, transmission of longer repeats to offspring results in an earlier onset of disease, where extensive somatic expansion in the striatum, the brain region primarily affected in HD, is proposed to accelerate disease pathology. Thus, understanding the mechanisms of trinucleotide repeat instability is a major interest. We have examined the role of oxidative DNA damage and base excision repair (BER) in somatic instability, which is tissue-selective and age-dependent. We show that oxidative DNA lesions abnormally accumulate at CAG expansions in a length-dependent, yet age- and tissue-independent manners, likely due to the secondary structures formed by CAG repeats that limit access of enzymes initiating BER. In addition, our data indicate that repair by BER enzymes of some of the accessible lesions results in somatic expansion when the ratio of FEN1 to POLβ is low, as found to occur in the striatum. Our results support BER enzyme stoichiometry as a contributor to the tissue selectivity of somatic CAG expansion in HD.

## Introduction

Huntington's disease (HD) is a neurodegenerative disorder caused by aberrant expansion of a CAG repeat tract within the coding sequence of the Huntingtin (*HTT*) gene, resulting in the production of a mutant protein with a toxic elongated polyglutamine (polyQ) stretch. This dominantly inherited disease, which shares the same mutation mechanism with eight other neurodegenerative disorders, is characterized by preferential and progressive degeneration of the medium-spiny neurons in the striatum. The HD mutation is unstable in germline and somatic cells, and CAG repeat expansion in both cell types has deleterious clinical consequences. Transmission of the mutation to offspring, in particular by fathers, is characterized by an expansion bias, leading to the phenomenon of anticipation [1]–[3], whereby the disease tends to worsen over successive generations due to the production of mutant huntingtin protein with an increased glutamine tract length that triggers earlier disease onset and increased disease severity. In addition, somatic CAG repeat expansion occurs in several tissues, including the brain. Interestingly, different brain regions are unequally affected [2],[4]; somatic instability is extensive in striatal neurons but very limited in cerebellar neurons, which are largely spared by the disease [5],[6]. It is therefore proposed that somatic expansion in the striatum and other target tissues, leading to the production of increasingly toxic mutant huntingtin proteins, accelerates HD pathology and acts as a disease-modifier [5]–[7]. Consistent with this hypothesis, an early mutant huntingtin phenotype is delayed in HD mice in which somatic instability is prevented by a deficiency in either the Msh2 or Msh3 mismatch repair (MMR) protein [8],[9].

Several studies support the notion that DNA repair contributes to somatic instability of CAG repeats, though other DNA-associated processes, such as replication, may also play a role [10]. In particular, at least two repair pathways appear to regulate *in vivo* somatic instability in the brain of HD mouse models. In addition to MMR, whose role is well documented [8],[9],[11],[12], base excision repair (BER), which is specialized in DNA base damage removal, has recently been implicated. Indeed, somatic CAG repeat instability was reduced in HD mice lacking the DNA glycosylase OGG1, an enzyme that initiates BER of 8-oxoguanine (8-oxoG) lesions [13]. Furthermore, the global level of oxidative DNA damage increased with age in the brains of HD mice, while OGG1 activity did not change, suggesting that the level of DNA oxidation at CAG expansions might underlie the age dependence of somatic instability [13]. *In vitro* biochemical and yeast studies have further suggested that other BER enzymes might modulate somatic instability. DNA polymerases, including DNA polymerase beta (POLβ), a central participant in most BER responses, can promote CAG repeat extensions *in vitro* [13]. In contrast, Flap Endonuclease-1 (FEN1), involved in the long-patch BER (LP-BER) subpathway, prevents CAG repeat expansion in yeast [14].

BER is characterized by a sequence of highly coordinated steps [15],[16], starting with damaged base recognition and removal by a DNA glycosylase, leading to formation of an apurinic/apyrimidinic (AP) site. In the brain, OGG1 appears to be the major glycosylase involved in removing endogenous lesions such as 8-oxoG from the genome [17]. Cleavage at the AP site is performed by the major AP endonuclease, APE1, which incises 5′ to the damage leaving behind a 3′OH and a 5′deoxyribose phosphate (5′dRP), which is subsequently removed by the dRP lyase activity of POLβ. A DNA polymerase then fills the resulting gap. In single-nucleotide BER (SN-BER), POLβ incorporates the missing nucleotide. However, when the 5′-terminal residue is refractory to POLβ lyase activity, repair proceeds via LP-BER. In this case, multiple nucleotides are incorporated by one of several DNA polymerases, including POLβ, POLδ or POLε, through a strand displacement mechanism. Subsequently, FEN1 is required to remove the 5′-flap structure formed during LP-BER synthesis. Interestingly, a number of studies suggest that BER enzyme activities vary with age and between tissues, indicating that the repair kinetics of oxidative DNA damage may be age- and tissue-dependent [18].

We sought to clarify the role of DNA damage and BER in the tissue specificity and age dependence of somatic CAG expansion in HD. First, we asked whether DNA lesions accumulated at CAG repeats, particularly in the striatum. Second, we asked whether BER proteins and activities displayed a specific or unique pattern of expression in the striatum. We have systematically analyzed DNA damage at CAG repeats, BER protein levels, and BER activities in both the unstable striatum, and in the mostly stable cerebellum of young and aged HD mice. Our results show that the stoichiometry of BER enzymes, rather than DNA damage levels, correlates with the tissue selectivity of somatic CAG expansion.

## Results

### Normal aging contributes to somatic CAG instability in R6 HD mice

Several studies have suggested that HD pathology contributes to neuronal aging. Specifically, oxidative stress and consequently oxidative DNA damage, which accumulates during normal aging, are elevated in HD neurons [19]. Thus, the “aging process” for HD neurons can potentially be envisioned as two separate components: (i) normal and (ii) disease-associated. We set out to determine the role of these two components in the process of somatic expansion. To this end, we compared various R6 transgenic mouse models of HD, which recapitulate several features of the human pathology, including somatic instability [6],[20]. R6/1 mice express the first exon of the human *HTT* gene with ∼125 CAG repeats and develop a progressive disease leading to death within approximately 8 months. The same transgene is expressed in R6/2 mice, yet is inserted in an alternate genomic location. R6/2–160 CAG and R6/2–100 CAG (spontaneously derived from R6/2–160 CAG) mice contain 160 and 100 CAG repeats, respectively. In both R6/2 lines, disease onset and progression is much more severe than in the R6/1 mice. Indeed, R6/2–160 CAG and R6/2–100 CAG mouse lines die at 3 and 4.5 months, respectively [21].

**Figure 1:**
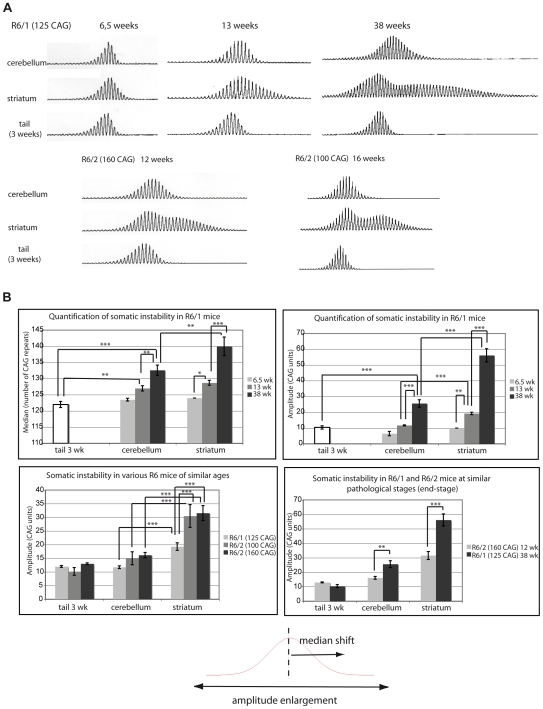
Comparison of various R6 mouse lines shows that natural aging contributes to somatic CAG expansion in HD mouse models. (A) Representative Genescan profiles showing CAG repeat size distribution from genomic DNA prepared from striatum or cerebellum of R6/1, R6/2 (160 CAG) and R6/2 (100 CAG) mice of various ages. The same transgene—except for CAG repeat length—is inserted in the different R6 mouse lines. One transgene copy is integrated into both the R6/1 and R6/2 lines, although integration sites are different [21]. For each mouse analyzed, the Genescan profile of the tail was also determined at 3 weeks to calculate the number of CAG repeats initially transmitted (corresponding to the peak with the highest fluorescent intensity). (B) Quantification of somatic CAG expansion in R6/1, R6/2 (160 CAG) and R6/2 (100 CAG), using either the median or the amplitude of the profiles as quantitative parameters. Comparison of mean medians (top left panel) and mean amplitudes (top right panel) between the striatum and the cerebellum of R6/1 mice with aging. Middle left panel. Comparison of mean amplitudes in striatum and cerebellum between R6/1, R6/2 (100 CAG) and R6/2 (160 CAG) mice at similar ages (12–14 weeks). Middle right panel. Comparison of mean amplitudes in striatum and cerebellum of R6/1 and R6/2 (160 CAG) at similar pathological stages, *i.e.* 38 weeks and 12 weeks, respectively. Mean values were calculated from 4 to 8 independent profiles (obtained from different mice). Error bars, sem; *, p<0.05; **, p<0.01; ***, p<0,001 (ANOVA followed by Newman Keuls test for post-hoc comparisons). Bottom. Somatic CAG instability leads to both amplitude enlargement of repeat size distribution and shift of the median towards longer alleles.

Somatic cell CAG instability was determined in R6/1 mice of various ages using standard PCR amplification and Genescan analysis from two brain regions that exhibit contrasting degrees of CAG instability, *i.e.* the striatum and the cerebellum (Figure 1A, top panel). To obtain a baseline reference for the number of CAG repeats, we analyzed tails from mice at 3 weeks of age, because the CAG repeat region from this tissue is mostly stable when taken from very young animals. As expected, quantification of instability showed that both the median and the amplitude of the Genescan profiles increased upon aging in the R6/1 striatum, but to a much lesser extent in the R6/1 cerebellum (Figure 1B, upper panels). As previously described, the profiles generated from aging striata, which display extensive somatic instability, were bimodal [6].

Comparison of 13 week-old R6/1 mice with R6/2–160 CAG and R6/2–100 CAG mice of similar ages, 12 and 16 weeks, respectively, showed that CAG repeat expansion was significantly higher in the striatum of R6/2 mice (Figure 1A and 1B, lower left panel). Thus, the process of somatic CAG instability was quicker in R6/2 mice than in R6/1 mice, regardless of the repeat length in the R6/2 mice, suggesting that disease-associated mechanisms and/or positional effects are involved in somatic expansion. However, when R6/1 and R6/2–160 CAG were compared at similar pathological stages (*e.g.* end-stages), we found that somatic instability was significantly higher in the striatum and the cerebellum of the R6/1 mice (Figure 1A and 1B, lower right panel). In agreement with previous results [6], these data indicate that normal aging contributes to somatic CAG instability.

### Global DNA damage does not correlate with tissue-specific propensity of somatic CAG instability

**Figure 2:**
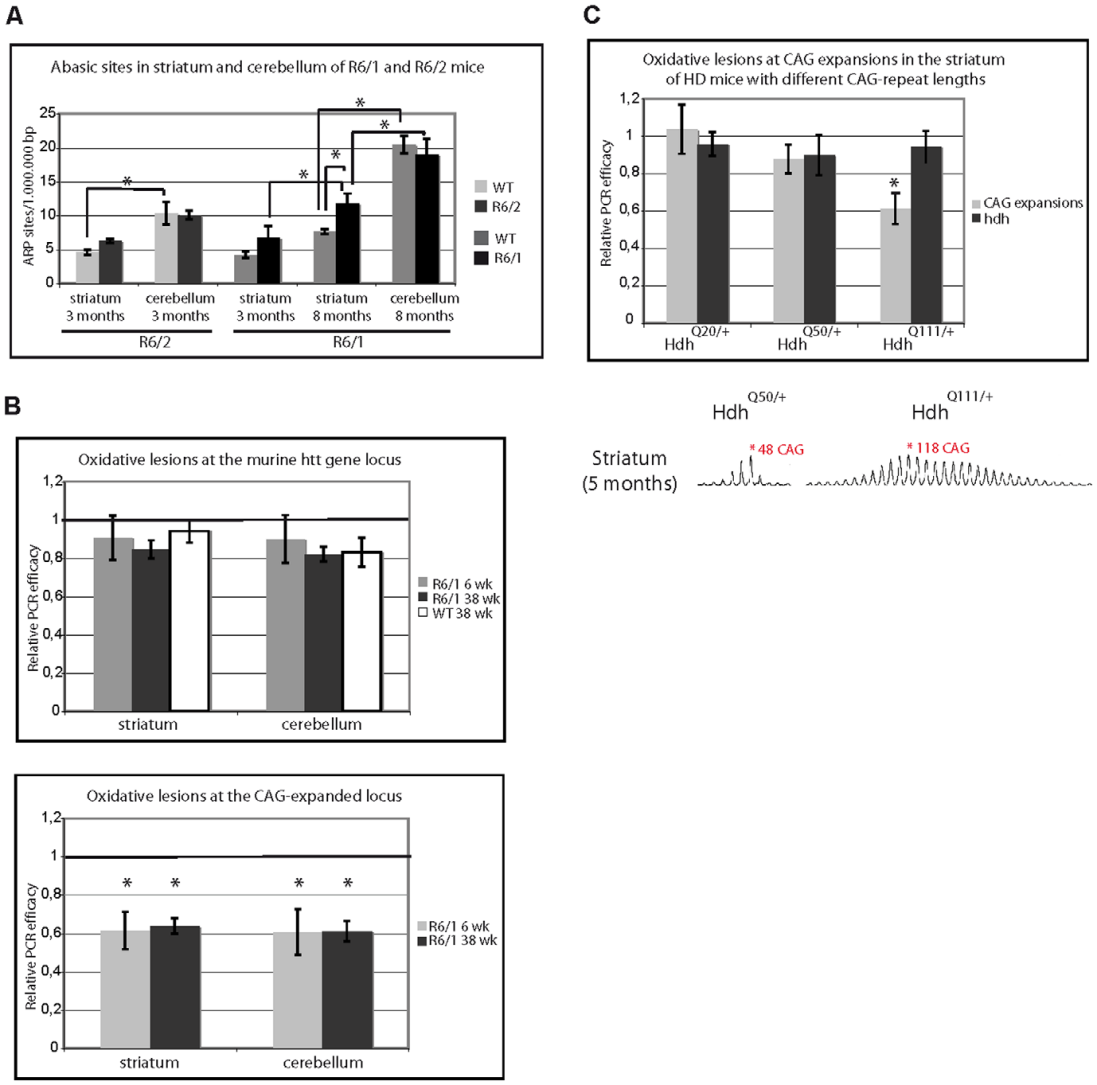
DNA damage accumulates at CAG expansions. (A) Quantification of AP sites from R6/1 or R6/2 mice, as well as their control littermates. AP sites were measured from striatum and cerebellum of 3 month-old R6/2 mice (at end-stage pathology) and from striatum and cerebellum of 3- or 8-month-old R6/1 mice (early- and end-stage pathology, respectively). 3 to 4 mice of each genotype and age were used. Error bars, sem; *, p<0.05; **, p<0.01; ***, p<0.001 (ANOVA followed by Newman Keuls test for post-hoc comparisons). (B) Oxidative DNA damage level at the murine *HTT* gene locus (*Hdh*) (top panel) and at the transgene locus (CAG-expanded locus) (bottom panel) in striatum and cerebellum of R6/1 mice at 6 or 38 weeks. Oxidative DNA damage levels at the murine *Hdh* gene locus were also determined from wild type littermate controls (WT) at 38 weeks. Ratios plotted in the graphs correspond to the Q-PCR–deduced DNA relative concentration of samples after Fpg treatment divided by the Q-PCR relative concentration of untreated samples. A ratio of 1 means that the specific gene locus tested did not accumulate oxidative DNA damage, while a ratio below 1 indicates that oxidative DNA damage accumulated. 3 to 4 mice of each genotype and age were analyzed. Error bars, sem; *, p<0.01 (Student's t-test). (C) Top panel. Oxidative DNA damage level at the CAG-expanded and *Hdh* loci in striatum of heterozygous knockin (KI) HD mice at 5 months of age with either 18, 48 or 115 CAG repeats. 3 mice of each genotype were analyzed. *, p<0.01 (Student's t-test). Bottom panel. Representative Genescan profiles of CAG repeat size distribution in the striatum of *Hdh^Q50/+^* or *Hdh^Q111/+^* mice.

It was recently proposed that DNA oxidation contributes to the age dependence of somatic CAG instability, due to damage accumulation in the brain during aging [13]. To clarify the role of DNA damage (and aging) in somatic expansion, we quantified AP sites within the whole genome in the striatum and cerebellum of R6/1 and R6/2 mice. AP sites are formed either spontaneously or as intermediates during repair of oxidized, deaminated or alkylated bases. AP sites increased with age in the striatum of R6/1 mice, with greater abundance at 8 months than at 3 months (Figure 2A). AP sites were also significantly higher at 8 months in the R6/1 striatum than in the striatum of littermate control animals, potentially reflecting increased oxidative stress or reduced repair in the HD mouse striatum. In contrast, the levels of AP sites detected in the cerebella of R6/1 were similar to those measured from control mice at 8 months (Figure 2A). To our surprise, we found that AP sites were significantly higher in the cerebellum *vs* striatum in age-matched animals for both the R6/1 and R6/2 lines (Figure 2A), indicating the level of DNA damage does not correlate with the tissue selectivity of somatic CAG instability in HD mice.

### Accumulation of DNA damage at CAG expansions in HD mice is neither tissue- nor age-dependent

We then asked whether DNA damage specifically accumulates at CAG expansions in the striatum of aging mice, as proposed by Kovtun *et al.* [13]. We evaluated the level of DNA oxidation at CAG expansions based on digestion of genomic DNA by the bacterial DNA glycosylase Fpg followed by real-time quantitative PCR (QPCR) as previously described [22]. The efficacy of PCR amplification was predicted to be reduced if the amplified DNA region contained Fpg sensitive sites, which includes several oxidative base lesions (*i.e.* 8-oxoG, 8-oxoadenine, fapy-guanine, methy-fapy-guanine, fapy-adenine, aflatoxin B_1_-fapy-guanine, 5-hydroxy-cytosine, 5-hydroxy-uracil), as well as AP sites, all of which are converted to strand breaks by the enzyme. As a control, we amplified a portion of the orthologous *HTT* gene in mouse (*Hdh*), which encompasses seven interrupted CAG repeats. We chose primers to amplify PCR fragments from the CAG-expanded transgene and the control *Hdh* locus that were equivalent in size and GC content. The sizes of the CAG-expanded and *Hdh* PCR fragments are 440 bp (corresponding to 120 CAG repeats) and 420 bp, respectively, with the GC content reaching 67.2% and 72.2% (Figure S1).

Given that the relative PCR efficiency was >0.8, the number of Fpg sensitive sites at the murine *Hdh* control locus appeared to be negligible in the striata and cerebella of both wild-type and R6/1 mice at 6 and 38 weeks of age, suggesting that spurious oxidation did not significantly affect the assay (Figure 2B, upper panel). In contrast, Fpg sensitive sites accumulated significantly at CAG expansions in the R6/1 mice, as revealed by a reduced relative PCR efficiency of ∼0.6 (Figure 2B, lower panel). Surprisingly, damage accumulation at CAG expansions was neither age- nor tissue-dependent, as the levels were similar in the striatum and cerebellum of young (6 weeks) and old (38 weeks) mice (Figure 2B).

We then measured Fpg sensitive sites in the striatum of mice with different CAG repeat lengths. For this purpose, we analyzed HD knockin mice heterozygous for the HD mutation and bearing either 18, 48 or 115 CAG repeats [23]. Accumulation of Fpg sensitive sites was CAG repeat length-dependent, as only mice with 115 CAG repeats displayed significantly reduced PCR amplification (Figure 2C). Thus, DNA damage at CAG expansions is not sufficient to trigger somatic instability, as accumulation of Fpg sensitive sites did not correlate with the age dependence or tissue selectivity seen in HD. The repeat length-dependence of DNA damage accumulation suggests that a build-up of presumably oxidative DNA lesions at CAG repeats might be dictated by the propensity to form alternate structures in DNA.

### DNA lesions at hairpin structures are inaccessible to OGG1 and APE1

**Figure 3:**
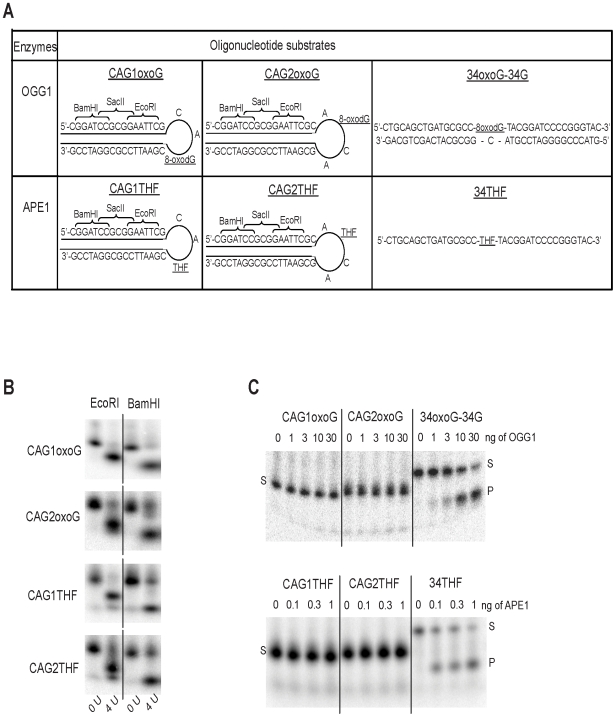
OGG1 and APE1 are unable to incise lesions at hairpin DNA structures. (A) Design of hairpin and control oligonucleotides. Hairpin oligonucleotides contain a 8-oxoG lesion or a THF lesion in 3-nt (CAG1oxoG or CAG1THF) or 4-nt (CAG2oxoG or CAG2THF) loop. The hairpin structured oligonucleotides contained *Bam*HI, *Sac*II and *Eco*RI restriction sites on the linear double stranded portion of the hairpin. (B) Digestion of hairpin oligonucleotides with *Eco*R1 or *Bam*H1 (4 U). Efficient digestion shows that the designed oligonucleotides adopt a stable hairpin structure. (C) Hairpin and control oligonucleotides were incubated with increasing amounts of OGG1 (top panel) or APE1 (bottom panel). S: substrate, P: product. Recombinant enzymes efficiently incised control, but not hairpin, oligonucleotides.

DNA sequences containing CAG repeats spontaneously form secondary structures such as hairpins [24],[25]. To assess the role of DNA structure in oxidative DNA damage accumulation at CAG expansion, we synthesized substrates mimicking hairpin structures formed by CAG sequences and containing either an oxidized base (8-oxoG) or an AP site analog (tetrahydrofuran, THF) at the tip of the hairpin. Two series of hairpin oligonucleotides were generated containing an 8-oxoG or THF modification at (1) the 3^rd^ position within a 3-nucleotide loop (referred to as CAG1oxoG and CAG1THF) or (2) the 2^nd^ position within a 4-nucleotide loop (referred to as CAG2oxoG and CAG2THF) (Figure 3A). Design of hairpin oligonucleotides included sites for *Eco*RI and *Bam*HI restriction enzymes (Figure 3A), which allowed for verification of hairpin structure formation. Digestion with either restriction enzyme revealed that the oligonucleotides formed the expected stable hairpin structure (Figure 3B). As additional controls, we designed linear oligonucleotides containing either an 8-oxoG lesion (referred to as 34-oxoG) or an AP site analog (referred to as 34THF). In contrast to OGG1, APE1 can incise single stranded oligonucleotides with varying efficiency, and thus, 34THF was used directly as a control [26],[27]. 34-oxoG, on the other hand, was annealed to a complementary oligonucleotide (34G) to form a control double stranded DNA substrate (34-oxoG/34G). To test whether OGG1 or APE1 were able to incise damage in hairpin substrates, increasing concentrations of human OGG1 or APE1 were incubated with the appropriate 5′-^32^P labeled oligonucleotide substrates. As shown in Figure 3C, no incision products were detected when using the aforementioned CAG hairpin substrates, even when OGG1 or APE1 concentrations were high (*i.e.* <2.5% product at 30 ng OGG1 or <3.5% product at 1 ng APE1). Conversely, both enzymes, even at low concentrations, efficiently incised their respective control substrates. Thus, OGG1 and APE1 are unable to efficiently incise hairpin substrates *in vitro*, supporting the idea that oxidative DNA lesions accumulate at CAG repeats because they can form secondary structures that are refractory to processing by BER enzymes.

### BER stoichiometry is different in the striatum and cerebellum of HD mice

**Figure 4:**
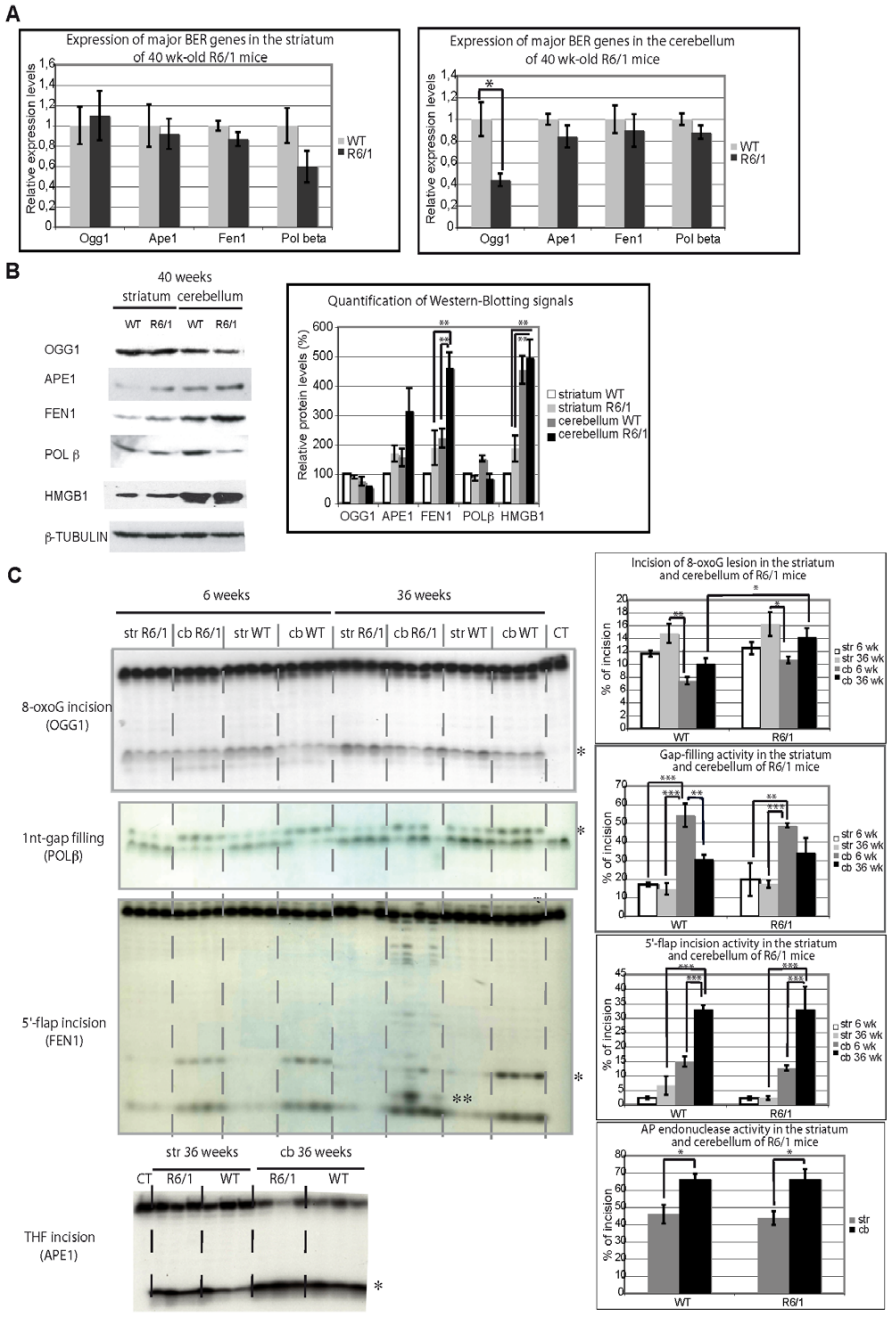
Analysis of BER mRNA, protein, and activity levels in striatum and cerebellum of R6/1 mice. (A) Real-time quantitative RT–PCR analysis of total RNA extracted from striatum (left panel) and cerebellum (right panel) of R6/1 mice and littermate controls (WT) at 40 weeks of age using primers specific for *Ogg1*, *Ape1*, *Fen1*, or *Polβ*. Error bars, sem. *, p<0.05 (Student's t-test). 3 to 4 mice of each genotype were used. (B) Left panel. Western blotting analysis of whole cell protein extracts from 40 week-old R6/1 or WT striata or cerebella. Cell extracts were prepared by pooling tissues from 4 animals. Right panel. Quantification of western blotting data. **, p<0.01 (ANOVA followed by Newman Keuls test for post-hoc comparisons). (C) Left panels. Activity analyses using a specific assay for each tested enzymatic activity (see Materials and Methods). Specific labeled substrates were incubated with striatal or cerebellar nuclear extracts prepared from R6/1 mice and control littermates, at both 6 and 36 weeks of age. *, normal incision or incorporation products; **, an additional incision product observed in FEN1 assay, when substrate was incubated with extracts from 36-week-old R6/1 cerebella, and included in quantification of the assay (see below). Right panels. Quantification of tested enzymatic activities in the striatal and cerebellar mouse extracts. The percentages of incision (likely OGG1, APE1, and FEN1) or incorporation (DNA polymerase), calculated by dividing the quantity of incised or elongated substrate by the total quantity of substrate, are represented on the graphs. *, p<0.05; **, p<0.01; ***, p<0.001 (ANOVA followed by Newman Keuls test for post-hoc comparisons).

Oxidative DNA damage is repaired by the BER pathway, which appears to be regulated in an age-dependent and tissue-specific manner [18]. We therefore asked whether the activity or stoichiometry of BER enzymes could contribute to the age dependence or tissue selectivity of somatic CAG instability in HD. We first determined mRNA expression levels of several major BER genes in the striatum and cerebellum of R6/1 HD and control mice at 40 weeks of age using quantitative RT-PCR. The BER genes that we tested were *Ogg1*, *Ape1*, *Fen1* and *Polβ*. Expression of most of these genes was similar between the transgenic and control mice, both in the striatum and cerebellum, except for *Ogg1*, which was downregulated 2-fold in the cerebellum of R6/1 mice (Figure 4A).

We next analyzed protein levels of several major BER enzymes by Western blotting (Figure 4B). The results showed that FEN1 was more abundant in the cerebellum than in the striatum of 40 week-old R6/1 mice. FEN1 was also produced at higher levels in R6/1 mice compared to control animals. APE1, POLβ and OGG1 levels were not significantly changed when comparing striatum *vs* cerebellum or R6/1 *vs* control animals. However, OGG1 tended to be lower in the cerebellum of R6/1 mice relative to the striatum, whereas APE1 appeared to be higher in the cerebellum of R6/1 mice. As a consequence, the stoichiometry of BER proteins was different between the cerebellum and striatum of R6/1 mice (Figure S3A). Using recombinant FEN1 and POLβ proteins as standards and Western blotting, we more precisely determined the stoichiometric ratio between FEN1 and POLβ in the striatum and cerebellum of R6/1 mice (Figure S3B). The results revealed that the FEN1/POLβ molar ratio was 1.4+/−0.3 in the striatum and 6+/−1.2 in the cerebellum corresponding to a 4-fold difference, thus supporting that the stoichiometry of BER enzymes is different in the striatum *vs* cerebellum of R6/1 mice, largely due to a significant disparity in FEN1 protein levels. We also analyzed the high-mobility group box 1 (HMGB1) protein, which was recently identified as a cofactor of BER that stimulates the activities of APE1 and FEN1 [28]. Interestingly, we found that HMGB1 protein was 2–3 fold higher in the cerebellum than in the striatum of R6/1 animals, suggesting that APE1 and FEN1 activities may be further activated by HMGB1 in the cerebellum of R6/1 mice. Finally, the level of BER enzymes and HMGB1 were similar in the cortex, another brain region characterized by substantial somatic expansion, to those in the striatum of R6/1 mice (data not shown), indicating that the stoichiometry of BER proteins might modulate the propensity of a given tissue for somatic CAG expansion.

### BER activities are less efficient in the striatum than in the cerebellum of HD mice

To evaluate further the role of BER in the tissue selectivity or age dependence of somatic CAG instability, we compared the enzymatic activities of the major steps of BER in extracts prepared from the striatum and cerebellum of R6/1 mice at both early- (6 weeks) and late- (36 weeks) stages. In addition, BER activities were compared between R6/1 and control littermate animals. Using standard oligonucleotide substrates (Figure S2), we performed *in vitro* biochemical assays allowing for evaluation of 8-oxoG lesion removal, gap filling, 5′-flap excision and AP endonuclease activities (Figure 4C). OGG1, POLβ, FEN1 and APE1 are most likely the main enzymes responsible for these activities, respectively, but other enzymes might contribute to the various activities as well. Strikingly, we did not observe significant differences between R6/1 mice and control animals regardless of the activity examined. Our results revealed that 8-oxoG incision tended to increase with age, both in the striatum and cerebellum of R6/1 and control mice, and to be higher in the striatum than in the cerebellum. In young animals (both R6/1 and controls), gap filling activity was significantly higher in the cerebellum compared to the striatum (by 2- to 3-fold). This difference disappeared at 36 weeks, due to an overall decrease in gap filling activity in the cerebellum (by 1.5- to 2-fold). AP endonuclease activity was slightly elevated in the cerebellum of both 36 week-old R6/1 and control mice (1.5 fold higher) when compared to the striatum. Most remarkably, 5′-flap incision activity was increased 5- to 10-fold in the cerebellum when compared to the striatum for both control and R6/1 mice. Furthermore, 5′-flap incision dramatically increased in the cerebellum upon aging, while it remained very low in the striatum. Finally, additional incision products were detected only in the cerebellum of 36 week old R6/1 mice, indicating that other exo- or endo-nuclease activities may be able to remove the 5′-flap structure in the cerebellum of aging transgenic animals.

Our results indicate that key steps implicated in BER, specifically downstream of 8-oxoG incision (OGG1) activity, are less efficient in the striatum than in the cerebellum of R6/1 mice. They also show that aging subtly modifies the stoichiometry of BER enzymes in a tissue-specific manner. Indeed, upon aging, 8-oxoG incision activity tends to increase both in the striatum and cerebellum, gap-filling activity decreases in the cerebellum and remains stable in the striatum, and flap endonuclease activity dramatically increases in the cerebellum, but remains stable, and low, in the striatum. These findings suggest that the LP-BER subpathway, which requires FEN1, is much less efficient in the striatum than in the cerebellum of R6/1 mice.

### Enrichment of POLβ at CAG expansions is tissue-specific

**Figure 5:**
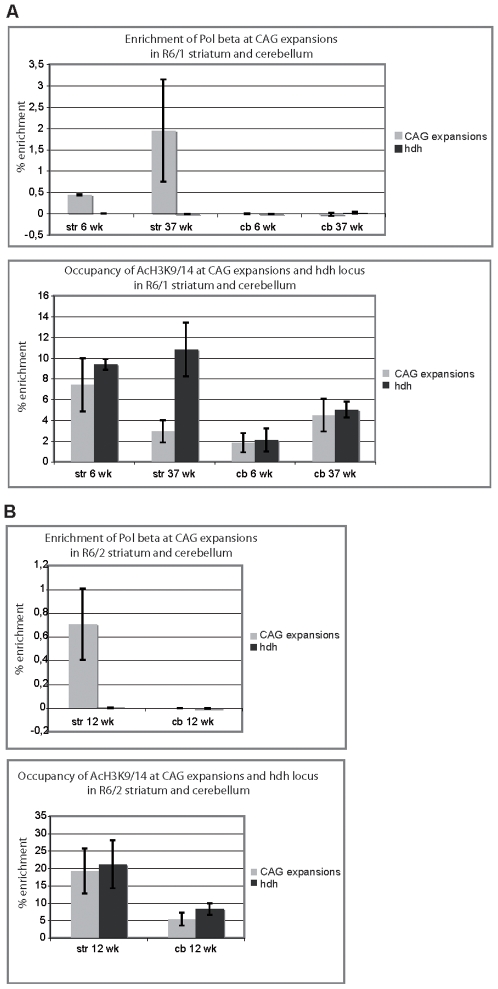
POLβ is specifically enriched at CAG expansions in the striatum but not in the cerebellum of HD mice. ChIP of CAG-expanded and *Hdh* loci from striatum and cerebellum of R6/1 (A) and R6/2 (B) mice using α-POLβ antibody (upper panels) and as control, α-AcH3K9/14 antibody (lower panels). (A) R6/1 mice at both 6 and 37 weeks of age and R6/2 mice at 12 weeks of age were analyzed. Each ChIP experiment was performed by pooling striata and cerebella from 2 to 4 mice. The values plotted on the graphs represent the mean values obtained from 3 to 4 independent experiments. The values correspond to percentage of enrichment, calculated as follows: % enrichment  =  (relative DNA concentration after ChIP with POLβ or AcH3—relative DNA concentration after ChIP with no antibody)/input. Relative DNA concentrations were measured using quantitative PCR. Error bars are sem.

To further interrogate the role of BER in somatic CAG instability *in vivo*, we performed chromatin-immunoprecipitation (ChIP) experiments. To set up conditions, we first used an antibody against acetylated K9/14 histone H3 (AcH3K9/14) that immuoprecipitates transcribed regions. ChIPed DNA was amplified using two primer sets encompassing the CAG-expanded transgenic region and a comparable portion of the mouse *Hdh* gene as a control. The results show that AcH3K9/14 is specifically and highly enriched at CAG expansions and at the *Hdh* locus in both the striatum and the cerebellum of R6/1 and R6/2 HD mice (Figure 5A and 5B, lower panels). This study indicates that our experimental conditions allowed for significant and specific detection of proteins bound to both CAG expansions and the *Hdh* gene. We then employed several antibodies against BER proteins. α-OGG1 and α-APE1 antibodies did not reveal any specific enrichment at either CAG expansions or at the *Hdh* locus (data not shown). However, we did find that POLβ was specifically enriched at CAG expansions in the striatum, but not in the cerebellum of R6/1 mice. Interestingly, this enrichment tended to increase with age (Figure 5A). POLβ was also specifically enriched at CAG expansions in the striatum of R6/2 mice (Figure 5B). Thus, enrichment of POLβ at CAG expansions was tissue-specific. These *in vivo* data support the concept that POLβ promotes somatic expansion [13].

### POLβ multi-nucleotide gap filling synthesis is modulated by the stoichiometry of POLβ and FEN1 during DNA damage repari of CAG repeat substrates

**Figure 6:**
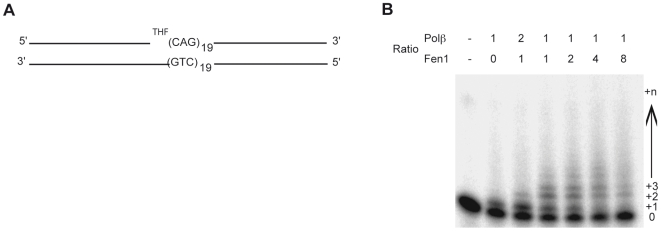
FEN1:POLβ stoichiometry modulates multi-nucleotide gap filling synthesis of POLβ during DNA damage repair of CAG repeat substrates. (A) CAG repeat substrate used for POLβ DNA synthesis and FEN1 flap endonuclease reactions. The substrate consists of a 23mer DNA primer strand, a gap, and a 76mer containing a 5′ tetrahydrofuran molecule (THF), 19 CAG repeats and 19 additional nucleotides (Top strands), both were annealed to a 100mer of complementary sequence. (B) Differential stoichiometric ratios of FEN1 modulate POLβ dependent synthesis of CAG repeat tracts. Reactions contained 2 nM POLβ and 1, 2, 4, 8, or 16 nM FEN1, respectively. Shown on top are POLβ/FEN1 ratios designed to recapitulate the ratios found in the specified HD mouse brain tissues. The position of the 23mer radiolabeled primer (0) or various extension products is indicated to the right.

Having shown that the FEN1/POLβ molar ratio is 1.4+/−0.3 in the striatum and 6+/−1.2 in the cerebellum of R6/1 mice (Figure S3B), that FEN1-like nuclease activity is 5- to 10-fold more elevated in the cerebellum (Figure 4C), and that POLβ is specifically enriched at CAG expansions in the striatum of HD mice (Figure 5), we suspected that the FEN1/POLβ ratio might be critical in determining the propensity of a given tissue to experience somatic CAG instability. To explore this possibility, we examined the effects of varying FEN1∶POLβ stoichiometry on POLβ DNA synthesis using a 100-mer substrate consisting of a 23-mer primer followed by a single nucleotide gap containing a 5′-abasic site analog (THF) and nineteen CAG repeats (Figure 6A). The effect of FEN1, at different molar ratios, on POLβ DNA synthesis activity was evaluated as follows. We employed purified POLβ at a single concentration, determined to give predominantly single nucleotide incorporation, and varied FEN1 to give 1∶2, 1∶1, 2∶1, 4∶1 and 8∶1 FEN1∶POLβ stoichiometries. We found that enhancement of POLβ multiple nucleotide incorporation, presumably due to strand displacement synthesis, was achieved at FEN1∶POLβ ratios of 1∶1 to 2∶1 and, to a lesser extent, 4∶1. Interestingly, at the 8∶1 ratio, POLβ multiple nucleotide synthesis was inhibited (Figure 6B). Under identical conditions, we observed no significant change in FEN1 flap endonuclease activity, although we did observe increasing 5′-flap endonuclease activity with increasing FEN1 protein (data not shown). These results support a model whereby BER enzyme stoichiometry affects the outcome of DNA damage processing, and as a consequence, potentially influences trinucleotide repeat instability.

## Discussion

**Figure 7:**
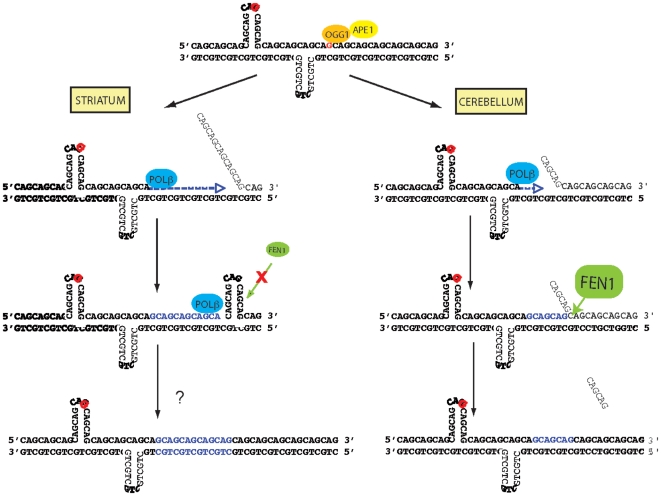
Model for somatic CAG expansion in HD. Pre-existing secondary DNA structures formed within CAG repeats prevent the accessibility of BER enzymes to oxidative DNA damage (red letters). When lesions become accessible, which occurs due to the dynamic nature of CAG repeats, repair can be initiated by OGG1. In the striatum, low flap endonuclease (FEN1) activity prevents cleavage of the 5′-flap structure generated by POLβ strand displacement activity, thereby leading to formation of an intermediate slipped strand structure. Processing of this intermediate structure, via an unknown mechanism, leads to somatic expansion. In contrast, in the cerebellum, high flap endonuclease (FEN1) activity permits efficient removal of the 5′-flap structure generated by POLβ strand displacement activity. Thus, no intermediate slipped strand structures are formed. Aging potentially worsens the situation because OGG1 activity tends to increase with age.

We have shown that oxidative DNA damage accumulates at CAG expansions in a CAG-repeat length-dependent, but age- and tissue-independent manner. Our data indicate that the DNA structure of CAG repeats likely contributes to this accumulation, as BER enzymes such as OGG1 and APE1 were unable to process lesions within hairpin configurations. In addition, comparing the striatum and cerebellum of HD mice, we have discovered a correlation between the propensity for somatic CAG expansion and BER protein stoichiometry and enzymatic activities. In particular, somatic instability correlates mainly with low flap endonuclease activity, as seen in the striatum. Our results also suggest that POLβ contributes to *in vivo* somatic expansion, as it is specifically enriched at CAG repeats in the striatum of aging animals. We thus propose a model whereby BER contributes to somatic CAG instability (Figure 7). In this model, accumulation of DNA damage at CAG repeats results from low accessibility of BER enzymes to lesions in pre-existing secondary DNA structures formed within CAG repeats. Repair of accessible lesions, which would arise due to the dynamic nature of trinucleotide repeat sequences, would be initiated by a protein such as OGG1 as proposed by Kovtun *et al.* [13] and potentially lead to somatic CAG expansion. This outcome would be particularly likely where there is poor cooperation between the strand displacement activity of POLβ and the 5′-flap excision activity of FEN1 during LP-BER.

The models proposed to explain disease-associated trinucleotide repeat instability involve specific stable secondary DNA structures. *In vitro* approaches based on substrates containing CAG/CTG repeats have shown that stable slipped strand DNA or hairpin structures form in a repeat length-dependent manner [24],[25]. Furthermore, three repair outcomes, including correct repair, escaped repair and error prone repair, have been observed for plasmid-based substrates that mimic slipped strand structures formed by CAG/CTG expansions, supporting the idea that processing of such structures by neuronal proteins can result in somatic expansion [29]. Although direct evidence is still lacking, several observations support that CAG/CTG expansions form secondary structures *in vivo* and the stability of these structures underlies the process of CAG/CTG repeat instability. First, longer repeats are more unstable in humans and mouse models of CAG/CTG repeat diseases [1],[23]. Second, CAG/CTG repeat sequence interruptions, which reduce the propensity to form slipped strand DNA structures, also prevent instability in mice and humans [30],[31]. Third, our data showing an extensive and repeat length-dependent accumulation of oxidative DNA damage at CAG expansions in HD mice imply that secondary structures form that are resistant to repair enzymes at the repeat locus. We in fact demonstrate that 8-oxoG and AP lesions, when located within the hairpin loop, cannot be efficiently incised *in vitro* by OGG1 and APE1 respectively. These results are in agreement with other studies showing that excision of 8-oxoG by OGG1 is paired-base-dependent and that the repair efficiency of some BER enzymes is affected by surrounding sequence contexts [32]. Thus, the probability that 8-oxoG lesions, an abundant endogenous DNA damage, are detected at CAG/CTG expansions by OGG1 should be significantly reduced if repeat expansions form secondary structures such as hairpins, as we indirectly demonstrate (in Figure 2B and 2C).

Our results showing that POLβ is enriched at CAG expansions, in combination with the results from Kovtun *et al.* [13] that provide evidence that OGG1 modulates the extent of somatic CAG instability, indicate that at least some of the oxidative lesions present at CAG/CTG repeats are (or become) accessible and processed by the BER pathway. This would imply that the secondary structures formed by CAG/CTG repeats are dynamic in nature. Several studies support that MMR, replication and transcription contribute to instability of CAG/CTG repeats, although the underlying mechanisms remain elusive [10]. These physiological processes induce chromatin remodeling and strand unpairing and, therefore, could provide a window for remodeling of secondary structures. While replication is unlikely to be involved in somatic instability in neurons, MMR and transcription could possibly interplay with BER at repeat expansions. During transcription, chromatin opens and the secondary structures in the transcribed strand have to be disrupted to allow RNA polymerase to proceed, only to re-form at a slightly different place. Thus, transcription could indirectly increase accessibility and the probability of repair of some oxidative DNA lesions by BER. The mechanism by which MMR contributes to somatic instability of CAG/CTG repeats is yet unknown. However, recent data indicate that functional MMR is required [33],[34], suggesting that MMR proteins not only bind, but also disrupt loops or secondary structures at CAG/CTG repeats. Whether BER cooperates with other DNA-associated mechanisms to promote somatic CAG instability is an intriguing possibility.

Using HD mice, we have found that the global level of AP sites was elevated in the mouse cerebellum, which displays little instability relative to the striatum (Figure 2A). In addition, abnormal accumulation of DNA damage at CAG expansions was similar in cerebellum and striatum and did not increase with age (Figure 2B). Thus, the propensity for somatic instability, which increases with age and varies between tissues, does not strictly correlate with levels of DNA lesions at CAG expansions, unlike the toxic oxidation cycle model proposed by Kovtun *et al.* [13]. We have found that the corresponding activity and relative stoichiometry of major BER enzymes, including POLβ, APE1 and FEN1 varied between the striatum and cerebellum and to a lesser extent with age (Figure 4 and Figure S3). In particular, FEN1 protein level and 5′-flap endonuclease activity were much lower in the striatum than in the cerebellum. We therefore propose that stoichiometry and the relevant activity levels of the corresponding BER enzymes, rather than the level of oxidative DNA damage at repeats, is crucial in determining the probability that repair of lesions at CAG expansions drives somatic expansion.

A 5′-flap endonuclease activity, such as that of FEN1, has for some time been implicated in models of instability for CAG/CTG repeats. In yeast, CAG/CTG instability is enhanced in strains defective for *rad27* (FEN1) in a repeat-length dependent manner [14],[35]. It has been hypothesized that flap structures formed at CAG/CTG repeats during replication or repair inhibit FEN1, because they form complex secondary structures. In agreement, *in vitro* results have shown that secondary structures, such as hairpins, reduce processing by FEN1 at CAG/CTG repeats in a length-dependent manner [36]. Ligation of the unprocessed flap to produce an expansion mutation is supported by a study in yeast showing that overexpression of *cdc9* (Ligase I) increases the rates of trinucleotide repeat expansion [37]. However, FEN1 haploinsufficiency did not change the extent of somatic instability in HD and DM1 mice, though intergenerational expansion tended to increase in the HD background [38],[39]. As complete inactivation of *Fen1* is lethal, HD and DM1 mouse models could only be examined in the *Fen1* heterozygous state. One cannot therefore exclude that a compensatory activity prevents exacerbation of repeat instability in a *Fen1^+/−^* background. By showing that FEN1 protein and 5′-flap endonuclease activity levels in the striatum and cerebellum are inversely correlated with the propensity for somatic expansion, we revive a role for FEN1 in preventing CAG repeat instability.

In BER, FEN1 is required to remove the 5′-flap structure generated during LP-BER strand displacement DNA synthesis [40],[41]. Interestingly, studies support that the strand displacement activity of POLβ is also crucial in LP-BER in neuronal cells [42]. Furthermore, *in vitro* experiments have shown that FEN1 strongly stimulates the strand displacement activity of POLβ and, reciprocally, POLβ stimulates FEN1 [43],[44]. It has been suggested that the functional interaction between POLβ and FEN1 controls incision product size in LP-BER: the tighter the cooperation, the shorter the product. Thus, cooperation between POLβ and FEN1 is believed to be essential to allow efficient repair of an oxidative lesion via LP-BER [43],[44]. Our results show that POLβ is enriched at CAG repeats in the striatum of R6/1 mice (Figure 5). Additionally, gap filling and flap endonuclease activities are much lower in the striatum than in the cerebellum (Figure 4), and the molar ratio of FEN1/POLβ proteins is decreased by about 4-fold in the striatum when compared to the cerebellum (Figure S3B). Additionally, we show that differential molar ratios of FEN1 influence DNA synthesis length within CAG repeat-containing substrates by POLβ *in vitro*, with protein stoichiometries consistent with those found in the striatum leading to increased synthesis and stoichiometries like those found in the cerebellum causing reduced nucleotide incorporation (Figure 6). Altogether, the findings suggest that poor cooperation between POLβ and FEN1, as apparently present in the striatum of R6/1 animals, would result in incorporation of long stretches of nucleotides by POLβ strand displacement activity and the formation of long 5′-flap structures that are generally resistant to FEN1 cleavage activity and precursors to somatic expansion (see Figure 7).

*In vitro* studies have shown that FEN1 is also stimulated by other BER partners, including APE1 [44]–[46], and by cofactors such as HMGB1 [28], which are both higher in the cerebellum when compared to the striatum (Figure 4). Thus, low APE1 and HMGB1 levels in the striatum might further contribute to the intrinsically low levels of flap endonuclease activity, thereby reducing the probability that structured 5′-flaps at CAG/CTG repeats are processed by FEN1. Alternatively, studies have shown that FEN1 possesses, in addition to its flap endonuclease activity, exonuclease and gap-dependent endonuclease activities, which can help process unusual 5′-flap structures, such as those generated by triplet repeat sequences during maturation of Okazaki fragments [47]. Interestingly, when assaying 5′-flap endonuclease activity (Figure 4C), we identified an alternative product of shorter size, present only in the cerebellum of 36 week-old R6/1 mice, suggesting that additional exonuclease or endonuclease activities exist in the cerebellum, but not in the striatum of old transgenic animals. These activities might help cerebellar neurons to remove the structured 5′-flaps formed at CAG repeats. Conversely, the low intrinsic endonuclease and exonuclease activities of FEN1 (or FEN1-related enzymes) in the striatum would lead to the persistence of structured flaps, thereby resulting in the generation of pre-expanded alleles, which could be processed through an error prone mode to facilitate somatic expansion as shown by Panigrahi *et al.* [29] (see Figure 7).

In conclusion, our results agree with a model that incorporates a role for oxidative DNA damage and BER in somatic CAG instability, at least in the context of HD. Our results suggest that, in the cerebellum, optimal cooperation between gap filling by POLβ and 5′-flap excision by FEN1 during the repair of oxidative lesions via LP-BER prevents (or at least limits) formation of slipped strand structures at CAG repeats. Conversely, in the striatum, poor cooperation between these two enzymes likely leads to the formation of complex intermediate structures, which, as shown by Panigrahi *et al.* [29], can then be processed through an error prone mechanism to foster somatic expansion.

Note. While this manuscript was under revision, two papers came out from Liu *et al.* [48] and Lopez Castel *et al.* [49] that support a contribution of poor coordination between specific enzymatic steps during DNA damage repair to somatic expansion of CAG/CTG repeats. The study of Liu *et al.* [48], based on *in vitro* experiments, indicates that dysfunctional coordination between POLβ and FEN1 during LP-BER triggers somatic expansion of substrates containing CAG repeats. Our study nicely complements these data by proposing that the lack of coordination between the two corresponding enzymatic steps of BER is correlated with the stoichiometric ratios of POLβ and FEN1 and is tissue-dependent. The study of Lopez Castel *et al*. [49], which is based on the use of mammalian cell lines impaired for Ligase I, extends this view by suggesting that coordination with the downstream ligation step is also crucial.

## Materials and Methods

### Mouse lines and breeding

Hemizygous R6/1 and R6/2 (160 CAG) and R6/2 (100 CAG) mice from the Jackson Laboratory were maintained on a mixed CBAxC57BL/6 genetic background [21]. *Hdh^Q111^*, *Hdh^Q50^* and *Hdh^Q20^* heterozygotes were maintained on a CD1 outbred genetic background [23]. The experiments were approved by the ethical committee C.R.E.M.E.A.S (Comite Regional d'Ethique en Matiere d'Experimentation Animale de Strasbourg).

### DNA and RNA extraction

The isolation of genomic DNA from mouse striatum and cerebellum for analysis of oxidative DNA damage was performed under conditions that minimize *ex vivo* oxidation artifacts according to the protocol developed by *Lu et al.* [22]. To this end, the silica-gel-membrane based DNeasy Tissue Kit (Qiagen) was used. Importantly, 50 µM of the free radical spin trap phenyl-tert-butyl nitrone (PBN, Sigma) was included in all buffers. High temperature and phenol use were avoided. DNA extracts were treated with RNase I. Striatum and cerebellum DNA extracted under these conditions was also used for CAG repeat sizing. Tail DNA for CAG repeats sizing was isolated using a standard protocol. Total RNA for CAG sizing and quantitative RT-PCR analysis was prepared using the RNeasy Mini Kit (Qiagen).

### CAG repeat sizing and analysis

CAG repeat size was determined by PCR amplification using the HEX labeled primer 31329 and primer 33934 previously described [20]. PCR reactions were performed using the expand high fidelity DNA polymerase (Roche), according to manufacturer's instructions. PCR reaction products were subsequently purified using the Nucleospin Extraction II Kit (Macherey-Nagel). Products were then analyzed using the ABI Prism 3100 DNA analyzer instrument and GeneScan and Genotyper softwares. Size calibration was performed by including ROX 500 or ROX 1000 (Applied Biosystems) with the analyzed PCR products. Amplitude of Genescan profile was determined by calculating the number of peaks above 10% of the maximum fluorescent peak intensity. From the amplitude value, we deduced the median peak.

### Quantitative amplification of CAG repeats

To quantitatively amplify CAG repeats from R6/1 and R6/2 DNA or RNA, we used the following protocol. DNA was amplified with primers 31329 and 33934, previously described [20] using the Herculase Hotstart DNA Polymerase (Stratagene). Concentrations of dNTP and primers were those recommended by the manufacturer. 8% DMSO was included in the reaction, as well as Sybr green (Molecular probe). The PCR reactions were performed and analyzed on a Light Cycler instrument (Roche). PCR cycling conditions were as follows: DNA was first inactivated for 3 min at 98°C, followed by 45 cycles consisting in 40 seconds at 98°C, 30 seconds at 60°C and 2 min at 72°C.

### Real-time RT–PCR analysis

Reverse transcription was performed on 1 µg of total RNA using SuperScriptII (Invitrogen) and random hexamers according to the manufacturer instructions. We performed PCR amplification of cDNA on a Light-Cycler instrument (Roche). PCR primers for detection of *Ogg1*, *Ape*1, *Polβ*, *Fen1*, *Hprt*, *Gapdh* and *36B4* are available upon request.

### Detection of ABASIC (AP) sites

Detection of AP sites was performed using the DNA damage quantification kit (Biovision), according to the manufacturer's instructions. The method is based on specific reaction of the Aldehyde Reactive (ARP) reagent with the open ring form of AP sites. Briefly, genomic DNA was treated with ARP tagged with biotin residues. AP sites in the DNA were then quantified using an avidin-biotin assay followed by a colorimetric reaction [50].

### DNA damage analysis

DNA damage at specific gene loci was assayed by cleavage of genomic DNA with bacterial formamidopyrimidine glycosylase (Fpg). Fpg is an AP-lyase that specifically excises 8-oxoG among other oxidized bases, and then creates a single strand break at the site of the abasic product. Quantitative real time PCR was used to determine the level of intact DNA at CAG repeats before and after DNA cleavage by Fpg. As a control, a portion of the murine *Htt* gene, which is similar in size and GC content to the CAG-expanded locus, was amplified. The ratio of PCR products after Fpg cleavage to those present in untreated DNA was used to determine the level of intact DNA. We followed the protocol described by Lu *et al.* [22], with the following modifications: Fpg from Sigma was used and incubated with genomic DNA for 1h at 37°C at a concentration of 0.2 µg Fpg/µg of DNA. These conditions, which were predetermined by an Fpg dose response curve and time course, allowed the reaction to reach a steady state. After inactivation at 60°C for 5 min, the Fpg reaction underwent an ethanol precipitation step. DNA was recovered in water and then submitted for quantitative PCR. Conditions for quantitative amplification of the CAG-expanded locus are described above. Quantitative amplification of the *Hdh* locus was performed using a commercial PCR master mix (Qiagen) with forward (5′-TCGAGTCGCTCAAGTCGTTT-3′) and reverse (5′-ACTTCGCAAACTGGGAACGG-3′) primers and PCR conditions were as follows: a first step of denaturation was performed at 95°C for 15 min, followed by 45 cycles consisting in a denaturation at 95°c for 30 seconds, hybridation for 30 seconds at 55°C and elongation for 1 min at 72°C.

### Western blotting

Striata and cerebella were dissected and homogenized in lysis buffer containing 50 mM Tris-HCl pH 8.0, 10% glycerol, 5 mM EDTA, 150 mM KCl, a cocktail of protease inhibitors (Roche) and 1% NP-40. The extracts were incubated for 15 min on ice and centrifuged for 20 min at 13000 rpm and 4°C. Supernatants were collected and analyzed on SDS-PAGE gels. Rabbit α-OGG1 (Abcam), mouse α-POLβ (Biovision), rabbit α-APE1 (Abcam), rabbit α-FEN1 (Santa Cruz or Abcam), rabbit α-HMGB1 (Abcam) and mouse α-β-tubulin (Chemicon) were used at 1∶1000 dilutions and revealed with appropriate α-rabbit or α-mouse peroxidase-conjugated secondary antibodies (Jackson immunoResearch Laboratories) and the ECL chemiluminescence reaction (Pierce or Millipore).

### OGG1 and APE1 activity at lesions located at hairpin structures

Hairpin DNA substrates with specific modifications (Figure 3A) were purchased from Midland Certified Reagent Company, Inc. (Midland, TX). [γ^32^P]ATP 5′ radiolabeled oligonucleotides were generated as described [51]. After annealing, the labeled oligonucleotides were purified from unincorporated [γ^32^P]ATP by using a Bio-Rad Micro Bio Spin P30 column. Briefly, after spinning the column at 1000 g for 1 min to remove packing buffer, the columns used for purification of the 5′-^32^P THF substrates were washed twice with OPT buffer (25 mM Mops, pH 7.2, 100 mM KCl, 1 mM MgCl_2_) and the columns used for 5′-^32^P 8-oxoG substrates were washed twice with NEB2 buffer (New England Biolabs). Columns were centrifuged as above for each washing step. The labeled oligonucleotides were then eluted through the appropriate column by centrifugation at 1000 g for 4 min. The hairpin structure was verified by incubation of the 5′-^32^P labeled oligonucleotides (0.2 pmol) with *Bam*HI or *Eco*RI (0 U to 4U; New England Biolabs) and subsequent electrophoresis on a 15% polyacrylamide urea denaturing gel. OGG1 incision assays were performed by incubating 5′-^32^P labeled 8-oxoG substrate (0.2 pmol) with hOGG1 protein (0 to 30 ng; New England Biolabs) in NEB2 buffer at 37°C for 15 min. APE1 incision assays were performed by incubating 5′-^32^P labeled THF substrate (0.2 pmol) with hAPE1 protein (0 to 1 ng) in OPT buffer at 37°C for 5 min. Reactions were inhibited by the addition of stop buffer (95% formamide, 20 mM ethylenediaminetetraacetic acid [EDTA], 0.5% bromophenol blue and 0.5% xylene cyanol), and then heated at 95°C for 5 min. Reaction products were resolved by 15% polyacrylamide urea denaturing gel electrophoresis and imaged using a Typhoon phosphoimager.

### DNA repair assays

Cell extracts were prepared essentially as described [52]. Briefly, frozen striata or cerebella were homogenized in 10 mM HEPES–KOH, pH 7.7, 0.5 mM MgCl_2_, 10 mM KCl, 1 mM DTT buffer and then centrifuged at 2000 g at 4°C for 10 min. The pellet was resuspended in 20 mM HEPES–KOH, pH 7.7, 0.5 mM MgCl_2_, 420 mM NaCl, 0.2 mM EDTA, 25% glycerol, PIC (Complete Protease Inhibitor Cocktail EDTA-free, ROCHE), 1 mM DTT and gently stirred at 4°C for 20–30 min to allow for efficient nuclear lysis. The suspension was centrifuged at 14000 g at 4°C for 15 min. The supernatant was dialyzed against 40 mM HEPES–KOH, pH 7.7, 50 mM KCl and 2 mM DTT buffer overnight at 4°C. Cell extract concentrations were determined using a Bradford assay. OGG1, APE1, POLβ and FEN1 assays were performed using oligonucleotide substrates described by [26], see Figure S2. 0.1 pmol of [γ^32^P]ATP 5′-radiolabeled oligonucleotides were incubated with 10 µg of cell extract and assay buffer (75 mM KCl, 25 mM MgCl_2_, 7,5 mM dNTPs, 3,125 mM HEPES–KOH, pH 7.7, 1% glycerol, 0.25 µM EDTA) at 37°C. Incubation times were optimized for each repair activity: 3 h, 1 h, 2 h and 50 min were used for 8-oxoG incision (OGG1), AP-endonuclease (APE1), gap-filling (POLβ) and flap endonuclease (FEN1) assays, respectively. The samples were then treated with 0.5% SDS and 0.8 mg/mL of Proteinase K, heated at 55°C for 15 min, purified by phenol-chloroform extraction and resuspended in solution by adding equal volume of 98% formamide, 10 mM EDTA, bromophenol blue and xylene cyanol buffer. Reaction products were resolved by 20% polyacrylamide urea denaturing gel electrophoresis and imaged on radiographic films. Images were captured with GeneSnap and quantified with GeneTool softwares on Syngene Chemigenus XE machine.

### POLβ DNA synthesis reactions in the presence of FEN1

2 nM of purified human DNA POLβ [53] was incubated with increasing concentrations of purified human FEN1 [54] (1 nM, 2 nM, 4 nM, 8 nM and 16 nM) and 200 fmol of substrate for 30 min at 37°C in 50 mM HEPES-KOH pH 7.5, 10 mM MgCl_2_, 0.5 mM EDTA, 2 mM DTT, 2 mM ATP, and 20 µM each dATP, dCTP, dGTP, and dTTP. Reactions were stopped by the addition of stop buffer consisting of 90% formamide and 20 mM EDTA, boiled for 5 min and then loaded onto a 15% polyacrylamide-urea gel and run at 300 V for 5 hrs.

### Chromatin immunoprecipitation (ChIP)

ChIP experiments were performed essentially as described [55]. For each ChIP experiment, striata and cerebellar from 2 to 4 transgenic mice were pooled, cut into small fragments, fixed by adding 37% formaldehyde to a final concentration of 1% and incubated for 10 min at room temperature. Cross-linking was stopped by addition of glycine to 0.125 M. Tissue fragments were washed three times with cold phosphate-buffered saline and treated with sonication buffer (50 mM HEPES pH 7.9, 140 mM NaCl, 1 mM EDTA, 1% Triton X-100, 0.1% sodium dodecyl sulfate, 0.1% Na-deoxycholate) containing protease and phosphatase inhibitors. Tissue was then homogenized, and lysates were sonicated to obtain DNA fragments of 200 to 1000 bp, as revealed by ethidium bromide staining of aliquots separated on agarose gels (Figure S4). Samples were centrifuged to pellet debris and an aliquot was taken for gel analysis and input. The soluble chromatin fraction was pretreated for 1 h at 4°C with protein A Agarose/Salmon Sperm DNA -50% slurry- (Millipore). Samples were then incubated overnight at 4°C with α-DNA POLβ ab194 (ChIP grade, Abcam) or α-AcH3K9/14 (Upstate) antibodies. Protein A Agarose/Salmon Sperm DNA was then added, and the mixture was incubated for 2 h at 4°C. Agarose beads were washed twice for 10 min with sonication buffer, twice for 10 min with wash buffer A (sonication buffer with 500 mM NaCl), twice for 10 min with wash buffer B (20 mM Tris-HCl, pH 8.0, 1 mM EDTA, à.25 M LiCl, à.5% NP-40, 0.5% Na-deoxycholate), and finally with Tris-EDTA (TE, pH 8.0). Immune complexes were eluted from the beads with 1% SDS in TE (pH 8.0) and protein-DNA cross-links were reversed by adding 200 mM NaCl and heating overnight at 65°C. After treatment with proteinase K for 2 h at 42°C, the samples were purified by phenol-chloroform-isoamyl alcohol extraction and precipitated with ethanol. One-sixth (for amplification of CAG expansions) to one-fifteen (for amplification of a fragment of the *Hdh* gene) of the immunoprecipitated DNA and 1% of the input DNA were quantified by real-time quantitative PCR (see above). Results are expressed relative to the amount of input DNA per ChIP.

## Supporting Information

Figure S1 Quantitative PCR amplification of CAG repeat locus from R6/1 mice. (A) Schematic representation of the HD transgene and the region surrounding exon 1 at the *Hdh* locus. Location of the primers used to amplify the CAG-expanded fragment and the *Hdh* control region is denoted by arrows. (B) Analysis of the GC content of the PCR fragment containing CAG expansions and of the PCR fragment located at the *Hdh* locus using DNA strider software. Both fragments are similar in size and GC content (around 70%). (C) Real time quantitative PCR amplification of CAG expansion from R6/1 striatum and cerebellum. Top left. Fusion profiles showing that primers 31329 and 33934 allow for amplification of a product specific to R6/1 mice. Bottom. Representative analysis of PCR amplification of CAG expansion from the striatum of R6/1 showing PCR is relative to the quantity of DNA doubling at each cycle (slope close to -3,3). Top right. Histogram showing that the relative DNA concentration calculated by the Light Cycler software is proportional to the initial quantity of DNA and similar between striatum and cerebellum.
(0.74 MB TIF)

Figure S2 Oligonucleotide substrates used to assess BER activities from mouse tissues. (A) Table showing the modified oligonucleotides used to assess the different steps involved in BER. Oligonucleotides containing an 8-oxodG and a tetrahydrofuran modification (THF) are used to assess glycosylase and AP-endonuclease activities, respectively. The gap-filling activity was assessed with two adjacent olionucleotides producing a 1-nucleotide gap. 5′-flap excision activity was evaluated using an oligonucleotide with a 10 nt flap. The enzymes that mainly carry out the corresponding reactions, i.e. OGG1, APE1, POLβ and FEN1, respectively, are shown on the left of the table. Due to functional redundancy other enzymes may contribute to the reactions. (B) Sequences of the oligonucleotides described above.
(0.24 MB TIF)

Figure S3 Stoichiometry of BER proteins is different in striatum and cerebellum of R6/1 mice. (A) Table showing the relative protein levels and activities of the designated BER proteins including OGG1, APE1, FEN1 and POLβ, in the striatum and cerebellum of R6/1 and control (WT) mice at 40 weeks of age. (B) Steady state levels of FEN1 and POLβ in the striatum and cerebellum of R6/1 mice were evaluated by western blotting using purified recombinant proteins corresponding to human FEN1 (42 kDa) and human POLβ (39 kDa), respectively. Top. 100 µg of whole cell extract (WCE) from the cerebellum of an R6/1 mouse were run on an SDS-polyacrylamide gel together with 15 ng and 150 ng recombinant FEN1 (left) or 15 ng and 150 ng recombinant POLβ (right) and detected with either α-FEN1 or α-POLβ antibodies. Band intensities were quantified and expressed as relative fold changes, which allowed calculation of FEN1:POLβ molar ratio. Bottom. WCE extracts from the striatum and cerebellum of two different 40 week-old R6/1 mice (numbered 1 and 2) were run on a gel and analyzed with α-FEN1 (rabbit), α-POLβ (mouse) and α-β-Tubulin (mouse). α-β-Tubulin was used to control sample loading. The same membrane was sequentially probed. Band intensities were quantified and the FEN1/POLβ molar ratio was estimated in the striatum and cerebellum of R6/1 mice. One representative set of detection with α-FEN1 and α-POLβ antibodies is shown. The extracts were loaded on gels and the antibody signal quantified 3 times independently. The mean and sem of the FEN1:POLβ molar ratios obtained after quantification of the 3 experiments are reported in the table.
(0.47 MB TIF)

Figure S4 Sonication of striatum and cerebellum extracts from R6 mice generates DNA fragments between 100 and 1,000 bp. DNA from striatum and cerebellum of R6/1 mice at 6 and 37 weeks of age was sonicated and analyzed by running aliquots on ethidium bromide stained agarose gels. DNA is sonicated to fragments below 1,000 bp.
(0.64 MB TIF)
