# Supplementary figures and images for: Stoichiometry of Base Excision Repair Proteins Correlates with Increased Somatic CAG Instability in Striatum over Cerebellum in Huntington's Disease Transgenic Mice
Source: PLoS Genet. 2009 Dec 4;5(12):e1000749. doi: 10.1371/journal.pgen.1000749 (PMC2778875; doi:10.1371/journal.pgen.1000749)

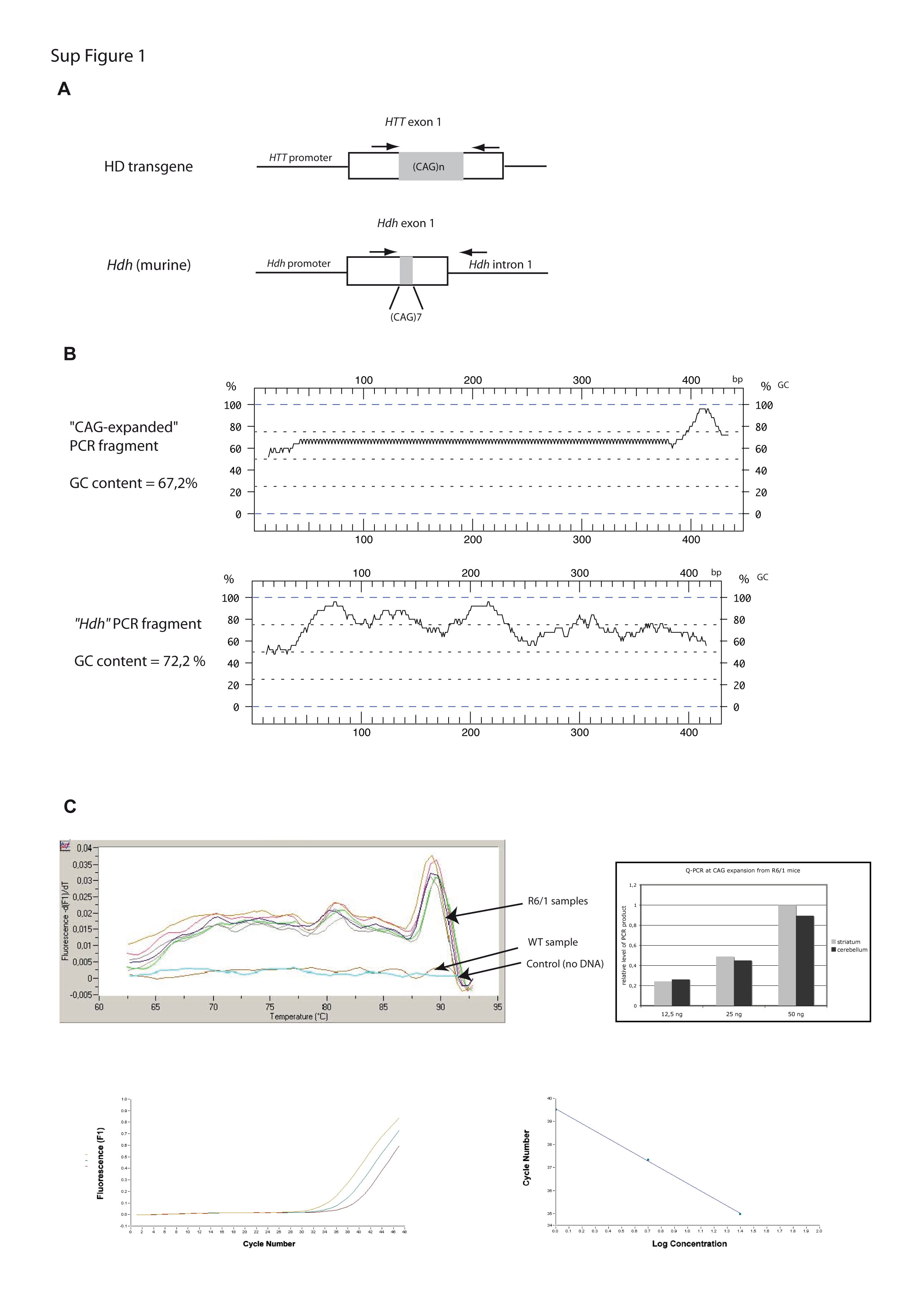

Supplement: Figure S1 — Quantitative PCR amplification of CAG repeat locus from R6/1 mice. (A) Schematic representation of the HD transgene and the region surrounding exon 1 at the Hdh locus. Location of the primers used to amplify the CAG-expanded fragment and the Hdh control region is denoted by arrows. (B) Analysis of the GC content of the PCR fragment containing CAG expansions and of the PCR fragment located at the Hdh locus using DNA strider software. Both fragments are similar in size and GC content (around 70%). (C) Real time quantitative PCR amplification of CAG expansion from R6/1 striatum and cerebellum. Top left. Fusion profiles showing that primers 31329 and 33934 allow for amplification of a product specific to R6/1 mice. Bottom. Representative analysis of PCR amplification of CAG expansion from the striatum of R6/1 showing PCR is relative to the quantity of DNA doubling at each cycle (slope close to -3,3). Top right. Histogram showing that the relative DNA concentration calculated by the Light Cycler software is proportional to the initial quantity of DNA and similar between striatum and cerebellum. (0.74 MB TIF) [file pgen.1000749.s001.tif]

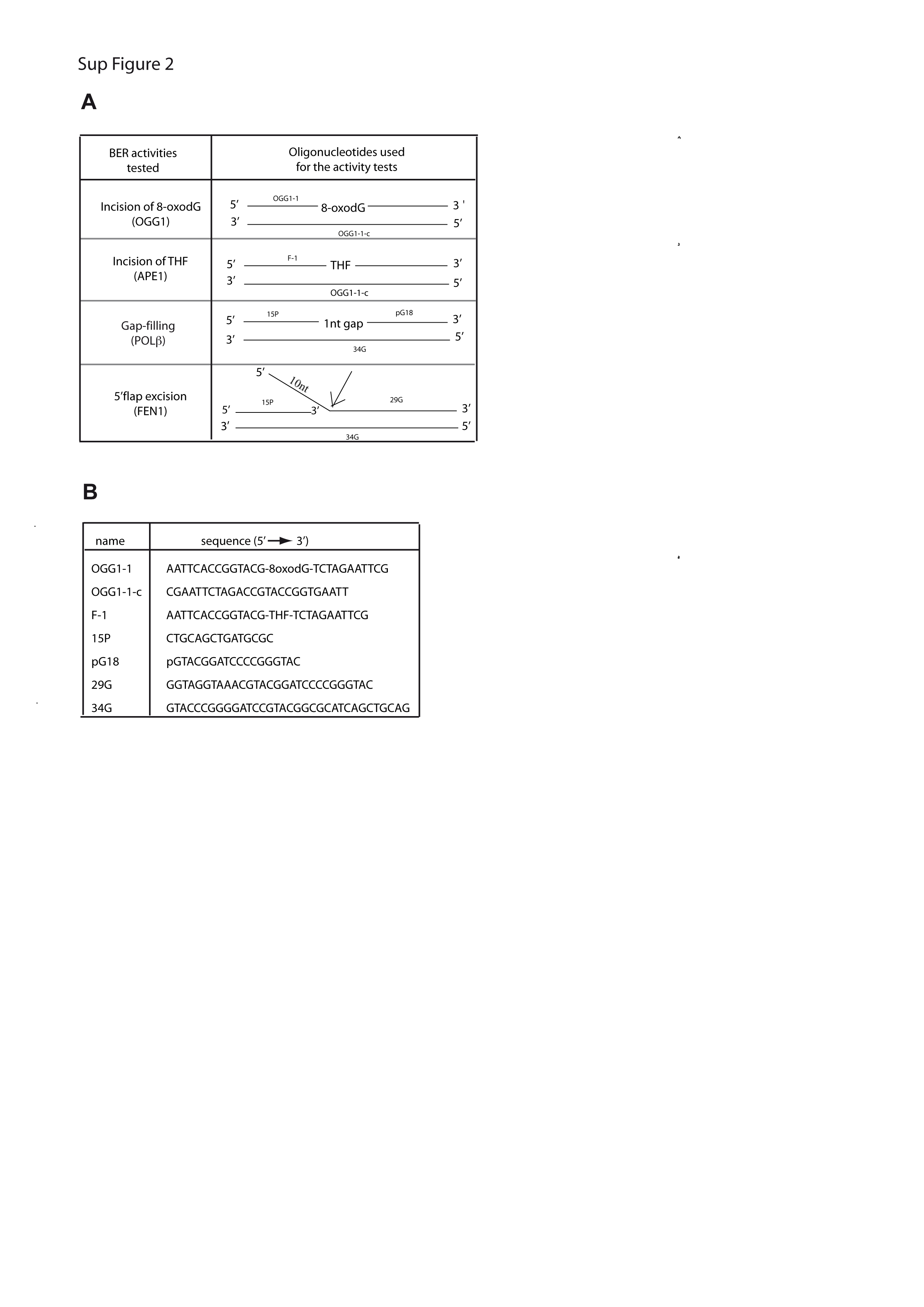

Supplement: Figure S2 — Oligonucleotide substrates used to assess BER activities from mouse tissues. (A) Table showing the modified oligonucleotides used to assess the different steps involved in BER. Oligonucleotides containing an 8-oxodG and a tetrahydrofuran modification (THF) are used to assess glycosylase and AP-endonuclease activities, respectively. The gap-filling activity was assessed with two adjacent olionucleotides producing a 1-nucleotide gap. 5′-flap excision activity was evaluated using an oligonucleotide with a 10 nt flap. The enzymes that mainly carry out the corresponding reactions, i.e. OGG1, APE1, POLβ and FEN1, respectively, are shown on the left of the table. Due to functional redundancy other enzymes may contribute to the reactions. (B) Sequences of the oligonucleotides described above. (0.24 MB TIF) [file pgen.1000749.s002.tif]

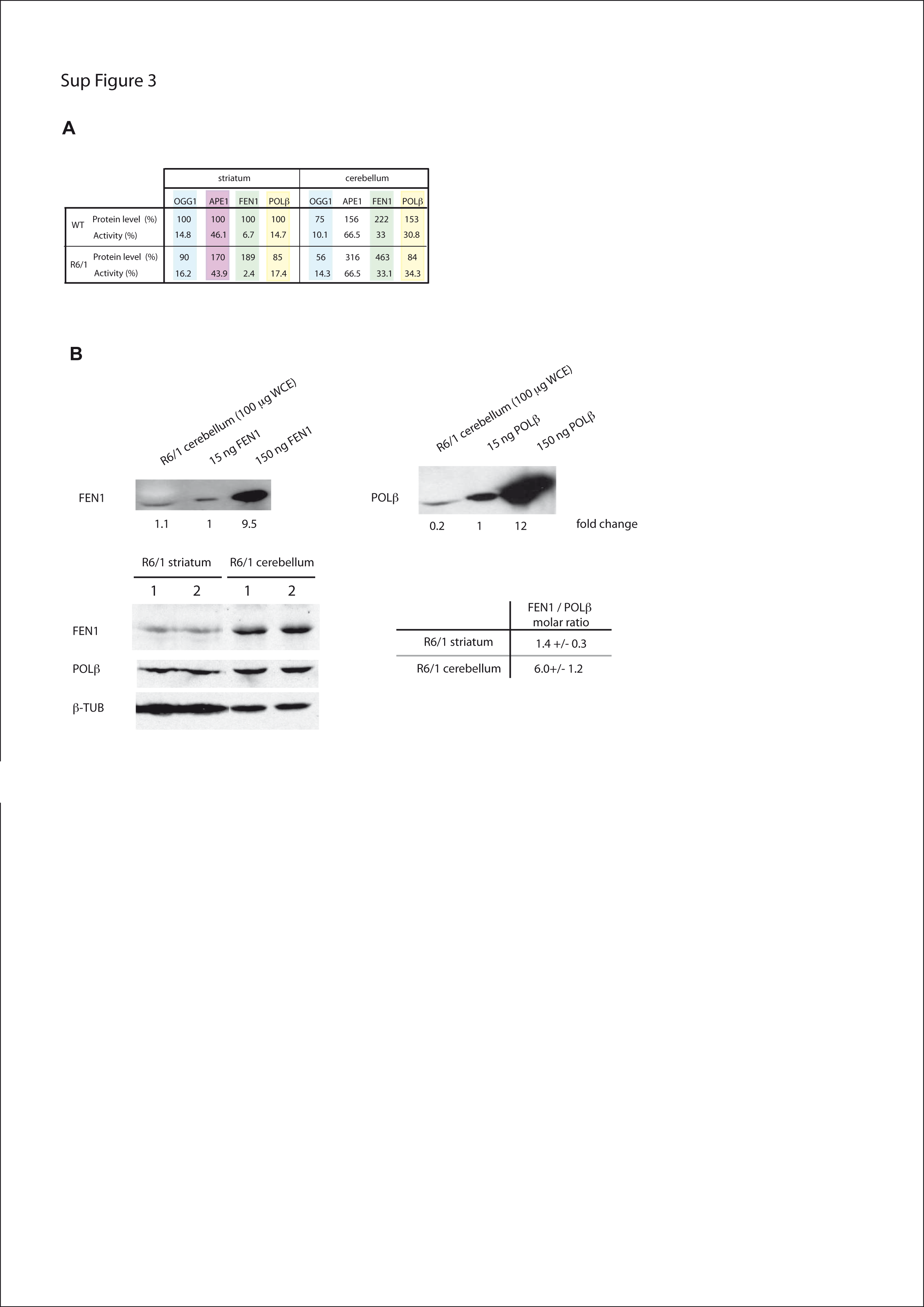

Supplement: Figure S3 — Stoichiometry of BER proteins is different in striatum and cerebellum of R6/1 mice. (A) Table showing the relative protein levels and activities of the designated BER proteins including OGG1, APE1, FEN1 and POLβ, in the striatum and cerebellum of R6/1 and control (WT) mice at 40 weeks of age. (B) Steady state levels of FEN1 and POLβ in the striatum and cerebellum of R6/1 mice were evaluated by western blotting using purified recombinant proteins corresponding to human FEN1 (42 kDa) and human POLβ (39 kDa), respectively. Top. 100 µg of whole cell extract (WCE) from the cerebellum of an R6/1 mouse were run on an SDS-polyacrylamide gel together with 15 ng and 150 ng recombinant FEN1 (left) or 15 ng and 150 ng recombinant POLβ (right) and detected with either α-FEN1 or α-POLβ antibodies. Band intensities were quantified and expressed as relative fold changes, which allowed calculation of FEN1:POLβ molar ratio. Bottom. WCE extracts from the striatum and cerebellum of two different 40 week-old R6/1 mice (numbered 1 and 2) were run on a gel and analyzed with α-FEN1 (rabbit), α-POLβ (mouse) and α-β-Tubulin (mouse). α-β-Tubulin was used to control sample loading. The same membrane was sequentially probed. Band intensities were quantified and the FEN1/POLβ molar ratio was estimated in the striatum and cerebellum of R6/1 mice. One representative set of detection with α-FEN1 and α-POLβ antibodies is shown. The extracts were loaded on gels and the antibody signal quantified 3 times independently. The mean and sem of the FEN1:POLβ molar ratios obtained after quantification of the 3 experiments are reported in the table. (0.47 MB TIF) [file pgen.1000749.s003.tif]

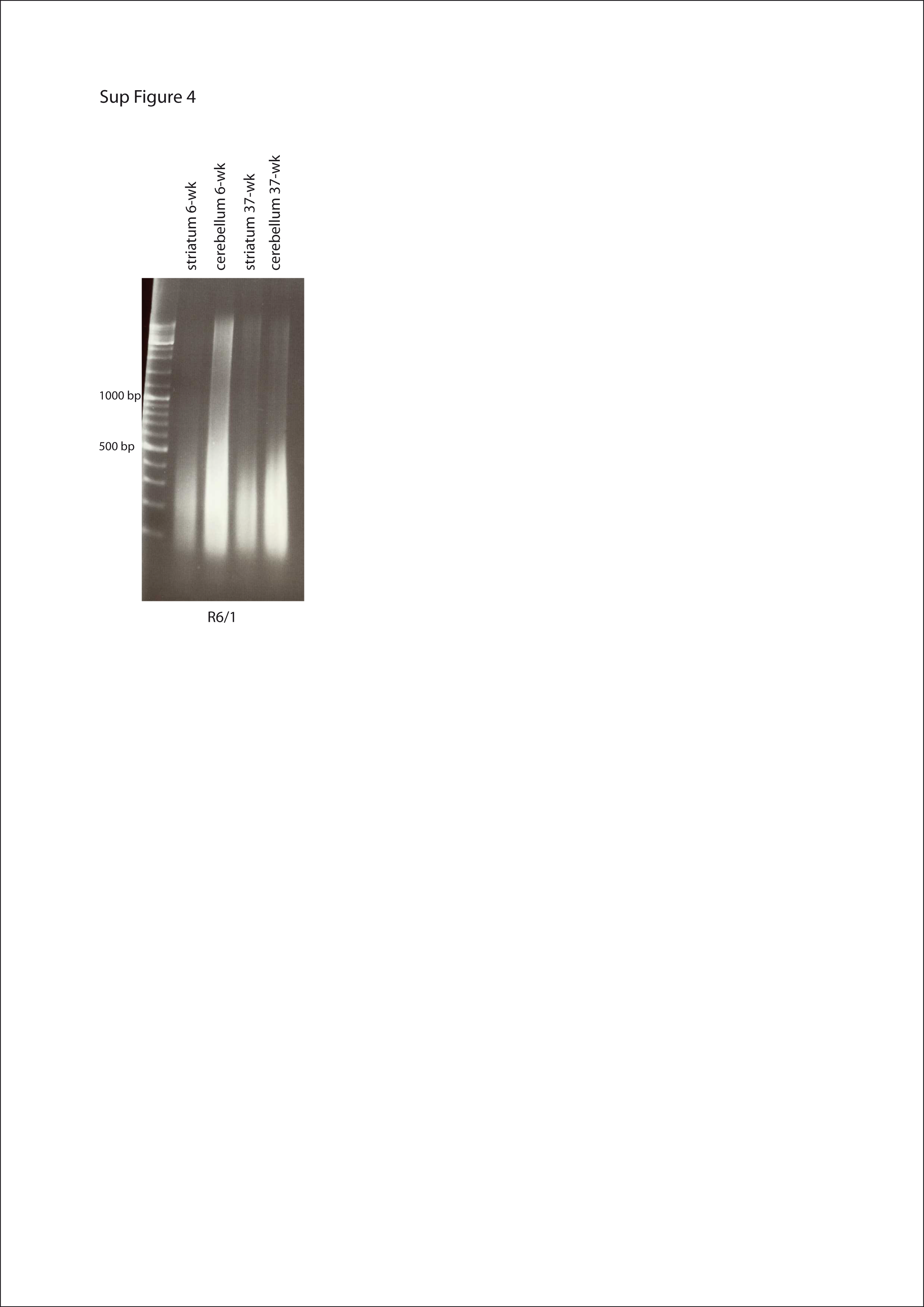

Supplement: Figure S4 — Sonication of striatum and cerebellum extracts from R6 mice generates DNA fragments between 100 and 1,000 bp. DNA from striatum and cerebellum of R6/1 mice at 6 and 37 weeks of age was sonicated and analyzed by running aliquots on ethidium bromide stained agarose gels. DNA is sonicated to fragments below 1,000 bp. (0.64 MB TIF) [file pgen.1000749.s004.tif]
